# Carbon ion radiotherapy: impact of tumor differentiation on local control in experimental prostate carcinomas

**DOI:** 10.1186/s13014-017-0914-9

**Published:** 2017-11-09

**Authors:** Christin Glowa, Peter Peschke, Stephan Brons, Oliver C. Neels, Klaus Kopka, Jürgen Debus, Christian P. Karger

**Affiliations:** 1 Department of Radiation Oncology, University Hospital Heidelberg, Im Neuenheimer Feld 400, 69120 Heidelberg, Germany; 2 Department of Medical Physics in Radiation Oncology (E040), German Cancer Research Center (DKFZ), Im Neuenheimer Feld 280, 69120 Heidelberg, Germany; 3 National Center for Radiation Research in Oncology (NCRO), Heidelberg Institute for Radiation Oncology (HIRO), Im Neuenheimer Feld 400, 69120 Heidelberg, Germany; 4 Heidelberg Ion Beam Therapy Center (HIT), Im Neuenheimer Feld 280, 69120 Heidelberg, Germany; 5 Division of Radiopharmaceutical Chemistry, German Cancer Research Center (DKFZ), Im Neuenheimer Feld 280, 69120 Heidelberg, Germany; 6 German Cancer Consortium (DKTK), Im Neuenheimer Feld 400, 69120 Heidelberg, Germany

**Keywords:** Carbon ion radiotherapy, Relative biological effectiveness (RBE), Prostate tumor, Hypoxia imaging

## Abstract

**Background:**

To summarize the research activities of the “clinical research group heavy ion therapy”, funded by the German Research Foundation (DFG, KFO 214), on the impact of intrinsic tumor characteristics (grading, hypoxia) on local tumor control after carbon (^12^C-) ion- and photon irradiations.

**Methods:**

Three sublines of syngeneic rat prostate tumors (R3327) with various differentiation levels (highly (-H), moderately (-HI) or anaplastic (-AT1), (diameter 10 mm) were irradiated with 1, 2 and 6 fractions of either ^12^C-ions or 6 MV photons using increasing dose levels. Primary endpoint was local tumor control at 300 days. The relative biological effectiveness (RBE) of ^12^C-ions was calculated from TCD_50_-values (dose at 50% tumor control probability) of photons and ^12^C-ions and correlated with intrinsic tumor parameters. For the HI-subline, larger tumors (diameter 18 mm) were irradiated with either carbon ions, oxygen ions or photons under ambient as well as hypoxic conditions to determine the variability of the RBE under different oxygenation levels. In addition, imaging, histology and molecular analyses were performed to decipher the underlying mechanisms.

**Results:**

Experimental results revealed (i) a smaller variation of the TCD_50_-values between the three tumor sublines for ^12^C-ions (23.6 - 32.9 Gy) than for photons (38.2 - 75.7 Gy), (ii) steeper dose-response curves for ^12^C-ions, and (iii) an RBE that increased with tumor grading (1.62 ± 0.11 (H) vs 2.08 ± 0.13 (HI) vs 2.30 ± 0.08 (AT1)). Large HI-tumors resulted in a marked increase of TCD_50_, which was increased further by 15% under hypoxic relative to oxic conditions. Noninvasive imaging, histology and molecular analyses identified hypoxia as an important radioresistance factor in photon therapy.

**Conclusions:**

The dose-response studies revealed a higher efficacy of ^12^C-ions relative to photon therapy in the investigated syngeneic tumor model. Hypoxia turned out to be at least one important radioresistance factor, which can be partly overridden by high-LET ion beams. This might be used to increase treatment effectiveness also in patients. The results of this project served as a starting point for several ongoing research projects.

## Background

Regarding the effectiveness of carbon ion beams, most systematic experimental investigations have been performed in vitro [1–3] or in normal tissue complication models in vivo [4, 5] and only very few quantitative data on the response of different tumor types to carbon ion beams are currently available [6–9]. As in treatment planning for photon radiotherapy, tumors are still considered as biologically homogeneous entities and the relative biological effectiveness (RBE) is computed by biomathematical models [10–12] to adjust for the dependencies of the RBE on linear energy transfer (LET) and dose. Biological response characteristics of the tumor are described by very few parameters neglecting the impact of additional tumor-associated biological factors on the RBE. However, any parameter that influences the tumor response differently for photons and ion beams is expected to influence the RBE. Therefore, to better understand the tumor response to ion beams, these dependencies have to be identified and assessed quantitatively in systematic preclinical experiments.

In previous studies, we determined the dose response curves for the anaplastic prostate carcinoma R2327-AT1 [13, 14] after 1, 2 and 6 fractions of photons and carbon ions, respectively, for the clinically relevant endpoint “local tumor control at 300 days”. It was found that the dependence on fractionation was much weaker than for normal tissue [4] resulting in smaller RBE-values at low fractional doses as compared to normal tissue. Although two other tumor types showed similar RBE-values at the same LET [7, 8], no systematic investigation of the RBE of tumors varying with respect to growth kinetic and differentiation status have been performed yet.

Within the translationally oriented clinical research group KFO 214 on heavy ion therapy, funded by the German Research Foundation (DFG), the radiation response of a well (-H), moderately (-HI) and poorly (-AT1) differentiated subline of the R2327 prostate carcinoma model was investigated in terms of dose-response curves for the endpoint “local tumor control at 300 days”. As it was found that the radiation responses of the three tumor lines differ much less for carbon ions than for photons, potential influence factors were further investigated by radiological imaging as well as on the histological and molecular level. Furthermore, the relationship between tumor microenvironment and local tumor control and its dependence on radiation quality was evaluated by dedicated irradiation experiments. This contribution gives a brief summary of the overall project and adds to previously published data [6, 15, 16].

## Methods

### Tumor model

Fresh tumor fragments of the syngeneic Dunning prostate adenocarcinoma sublines R3327-H, -HI and -AT1 [17] were implanted subcutaneously into the distal thigh of male Copenhagen rats (weight 180-200 g, Charles River Laboratories, Wilmington, Massachusetts, USA). During irradiation of H- and HI-tumors, rats were always kept under inhalation anesthesia with a mixture of 2.5% sevoflurane (Abbott, Wiesbaden, Germany) and oxygen at 2 l/min using an inhalation mask. For AT1-irradiations, animals were anesthetized with an intraperitoneal injection of Ketamine hydrochloride (125 mg/kg, Pfizer Deutschland, Berlin, Germany) mixed with Xylazine hydrochloride (20 mg/kg, Bayer HealthCare, Leverkusen, Germany) and breathed air [13]. Imaging studies were performed with 3-3.5% sevoflurane and 1 l/min oxygen. All experiments were approved by the governmental review committee on animal care, and animals were kept under standard laboratory conditions.

### Irradiation setup

The general experimental setup has been described previously [6, 13, 14]. Briefly, for tumor irradiations, rats were placed in a special device for accurate positioning. Tumors of two different sizes were irradiated: Small tumors with a mean diameter at treatment of 10.5 mm (range 9.0 to 12.0 mm) were irradiated with carbon ions at the center of a single 20 mm SOBP (dose-averaged LET in the tumor: 75 keV/μm, range 64-96 keV/ μm) having a field diameter of 18 mm (90% isodose). Large tumors had a mean diameter at treatment of 16.5 mm (range 15.5 to 18.5 mm) and were irradiated either with carbon or oxygen ions (^16^O-ions) at the center of a single 30 mm spread-out Bragg-peak (SOBP) (dose-averaged LET in the tumor: 65 keV/μm, range 52-91 keV/μm for carbon and 101 keV/μm, range 82-142 keV/μm for oxygen ions, respectively) having a field diameter of 25 mm (90% isodose). The range of the ions was adjusted by a polymethyl methacrylate (PMMA)-bolus of appropriate thickness. A second PMMA-plate was positioned behind the tumor.

Photon irradiations were performed under identical conditions using a single 6 MV beam of a linear accelerator (Siemens Artiste, Erlangen, Germany) and a PMMA-bolus in front and behind the tumor. Irradiation fields were produced with a cylindrical collimator for the small tumors (90% isodose: 15 mm) and with a multi-leaf collimator for the larger tumors (90% isodose: 24 mm), respectively.

### Dose response studies

For small tumors, dose response experiments were performed for all three tumor-sublines (AT1, HI and H) with either 1, 2 or 6 fractions using increasing dose levels of either carbon ions or photons. In total, this experimental series contained 859 animals (374 for carbon ions and 405 for photons) including 80 sham-treated controls.

In a second series, large tumors of the HI-subline were treated with single doses under oxic as well as under hypoxic conditions using increasing dose levels of either carbon ions, oxygen ions or photons. Hypoxic conditions were realized by clamping the tumor-supplying artery 10 min before and during treatment. In total, this experimental series contained 280 animals (45/44 for carbon ions, 37/36 for oxygen ions and 47/48 for photons under oxic/hypoxic conditions); 23 sham-treated animals served as controls.

Following irradiation, tumor volume was measured twice weekly in both experimental series using a caliper. Primary endpoint was local tumor control at 300 days, defined as no detectable tumor regrowth. As the H-subline exhibited residual nodules, they were harvested and analyzed histologically for fibrosis (Hematoxylin/Eosin; H&E) and proliferation 5-bromo-2′-desoxyuridine (BrdU). A fibrotic pattern without proliferation was considered as secondary endpoint for locally controlled H-tumors.

For the primary endpoint, actuarial control rates were calculated and the logistic dose-response model was fitted using the maximum likelihood fitting procedure of the software STATISTICA (version 10.0, Statsoft Inc., www.statsoft.com) (see [6] for details). For the secondary endpoint, no actuarial approach was required as surviving tumor cells were directly detected with a proliferation marker. For both endpoints, the RBE was calculated as the ratio of the TCD_50_-values (dose at 50% tumor control probability) for photons and ^12^C-ions.

### Positron-Emission-Tomography (PET)

Dynamic PET measurements with different radiofluorinated 2-nitroimidazole derivatives on a patient scanner (Biograph™ mCT, 128 S, Siemens, Erlangen, Germany) were performed to characterize the hypoxic status of small (0.8 ± 0.5 cm^3^) and very large (4.4 ± 2.8 cm^3^) H-, HI- and AT1-tumors prior to irradiation. For this, 15-53 MBq of [^18^F]fluoromisonidazole ([^18^F]FMISO) were injected into the tail vein of the animals and PET images were recorded over a time period of 60 min using a 28-frame protocol (for details, see [16]). In total, this study included 30 tumors (10 AT1, 12 HI and 8 H).

Additional static measurements in 12 HI-tumors (diameter 16 mm) were performed on a PET/CT (Inveon Micro-PET/SPECT/CT, Siemens Medical Solutions, Knoxville, USA) before and 2, 9, and 21d after carbon ion or photon irradiation, respectively. In these measurements, 38-52 MBq [^18^F]fluoroazomycin arabinoside ([^18^F]FAZA) were administered into the tail vein and images were evaluated at 2 h post-injection.

### T1-weighted dynamic contrast enhanced magnetic resonance imaging (DCE-MRI)

T1-weighted DCE-MRI measurements were performed in 17 small HI-tumors before as well as 3, 7, 14 and 21 days after single doses (isoeffective doses 18 Gy ^12^C-ions vs. 37 Gy photons and 37 Gy ^12^C-ions vs. 75 Gy photons, respectively) using a clinical 1.5 T MRI (Symphony, Siemens, Erlangen, Germany) together with an in-house built small animal coil. Irradiations were carried out either with carbon ions or photons using the same absorbed as well as the same RBE-weighted doses. Each animal had a sham-treated tumor on the contralateral side as internal control.

A T2-weighted turbo spin echo sequence (TR 3240 ms, TE 81 ms, slice thickness 1.5 mm, pixel size 0.35 mm) was used to position the image slice of the DCE-MRI measurement (TR 373 ms, TE 1,67 ms, slice thickness 4.5 mm, pixel size 0.99 mm) at the center of the tumor. 30 s after starting the DCE-MRI measurement, 0.1 mmol/kg Gd-DTPA (Magnevist^®^, Bayer Healthcare Pharmaceuticals, Berlin, Germany) was injected into the tail vein. Tumor volume and the kinetics of the contrast agent were analyzed using the in-house software “Medical Imaging Interaction Toolkit” (dkfz, Heidelberg, Germany [18, 19]).

### Doppler-Ultrasound imaging

Ultrasound imaging was performed for 16 small HI-tumors from different dose-groups of the carbon ion and photon single fraction dose-response studies using a Power Doppler Ultrasound of 30 MHz and the RMV-704 transducer (slice thickness 200 μm, VEVO770, VisualSonics, Toronto, Canada). Animals were measured before and weekly or 2-weekly after irradiation.

### Flow cytometric analysis

DNA-index and cell cycle distribution as well as potential surface stem cell marker of untreated tumors were identified with flow cytometry. Single cell suspensions obtained from frozen tissue were incubated with 2.1% citric acid including 0.5% tween 20 and shaking for 20 min at room temperature. Afterwards, 700 μl of the cell suspension supernatant were transferred into a vial, containing 4 ml phosphate buffer (Na_2_HPO_4_ 7.1 g/100 ml dH_2_O, pH 8.0) with 2 μg/ml 4′,6-diamidino-2-phenylindole (DAPI) and analyzed on a PAS II flow cytometer (PARTEC, Münster, Germany). For details see [15]. Cryo-preserved tumor tissue was prepared as single cell suspension using isolation buffer. Afterwards cells were stained for CD24-PE, CD44-FITC, CD133-PE, CD326-FITC, cytokeratin 5/8 and 19 labelled with an Alexa Fluor 488 secondary antibody and measured in the Galaxy pro Flow cytometer (PARTEC, Münster, Germany). Flow cytometric analysis was confirmed with staining of cryo-preserved and FFPE tumor tissue (for details, see [15]).

### Tumor induction analysis via limiting dilution assay

CD24^+^/CD45^−^ and CD24^−^/CD45^−^ untreated AT1-, HI- and H-tumor cells were enriched and sorted (FACS Aria, BD, Heidelberg, Germany) from freshly prepared tumor tissue. 500.000 CD24^−^/CD45^−^ cells and various cell numbers between 10 to 200.000 CD24^+^/CD45^−^ cells were injected in a Matrigel suspension (BD, Heidelberg, Germany) subcutaneously into the right and left thigh of animals. The tumor induction was monitored for 300 days.

### Histological and molecular studies

Before and at several time points after single dose irradiation (8 h, 18 h, 72 h, 7 d, 14 d, 21 d) tumor tissue was cryo-preserved, cut into 7 μm cryo-sections (Mikrom HM560, Thermo Fisher Scientific, Dreieich, Germany) and fixed in methanol/acetone for immunofluorescence stainings. Alternatively, formalin-fixed paraffin-embedded (FFPE) tissue was processed with the Microtom (Microm STS Section-Transfer-System, Thermo Fisher Scientific, Dreieich, Germany) and used for H&E staining.

To analyze the secondary endpoint in the H-tumor, cryo-preserved sections of the residual nodules were stained for proliferating cells using a BrdU antibody (Roche Diagnostics, Mannheim, Germany), which was injected intraperitoneally (100 mg/kg, Sigma-Aldrich, Taufkirchen, Germany) prior to sacrificing the animal. Vessel architecture (CD31), pericytes (smooth muscle actin) and perfusion as well as hypoxic fraction (pimonidazole) was stained using published protocols [6, 16].

For gene expression analysis, HI-tumor tissue was minced in liquid nitrogen using a Potter S with a Teflon tube extruder (B. Braun, Melsungen, Germany) and RNA was extracted immediately with the NucleoSpin® RNA L Kit (Macherey-Nagel, Düren, Germany). RNA-quantity (NanoDrop® ND-1000 Peqlab, Erlangen, Germany) and quality (Agilent RNA 6000 Nano Kit and Agilent Bioanalyzer 2100, Agilent, Waldbronn, Germany) were verified. Gene expression profiling was performed according to the manufacturers’ protocol (Agilent) using the Whole Rat Genome Kit 4x44k, Low Input Quick Amp Labeling Kit One-Color, gene expression hybridization Kit, RNA-Spike In Kit One-Color, SSPE washing buffer and stabilization and drying solutions.

## Results

### Dose-response studies for three different sublines

**Fig. 1:**
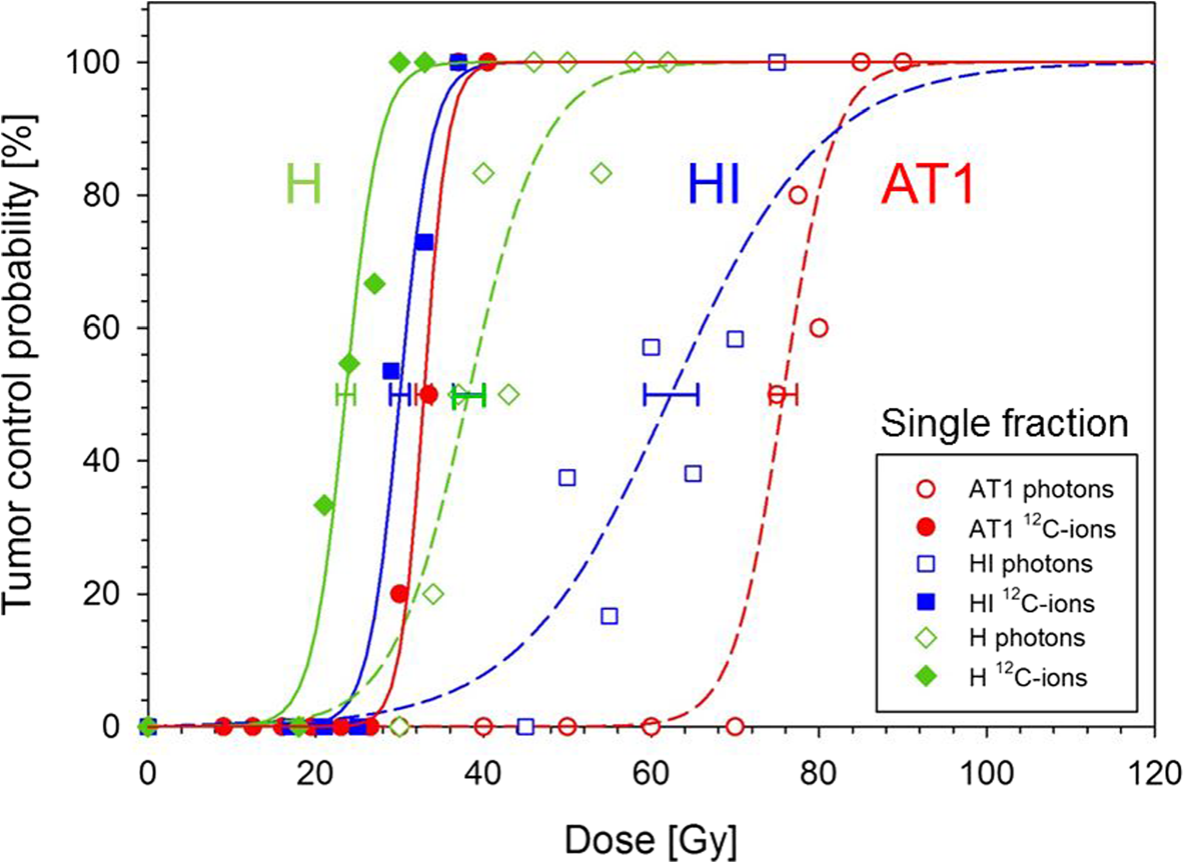
Dose-response curves of three sublines of the R3327 prostate carcinoma after a single fraction of photons (dashed lines) and ^12^C-ions (solid lines) for the endpoint local tumor control at 300 days, respectively. The uncertainty (1 SD) of TCD_50_ is indicated

Figure 1 displays the dose-response curves for the three tumor sublines after single doses of carbon ions or photons, respectively, using the primary endpoint “local control”. The TCD_50_-values were 75.7 ± 1.6 (AT1), 62.4 ± 3.2 (HI) and 38.2 ± 1.8 (H) for photons and 32.9 ± 0.9 (AT1), 30.0 ± 1.1 (HI) and 23.6 ± 1.1 (H) for carbon ions, respectively. The corresponding RBE-values were found to be 2.30 ± 0.08 (AT1), 2.08 ± 0.13 (HI) and 1.62 ± 0.11 (H).

Mean tumor regression (complete tumor volume reduction) times for the AT1-, HI- and H-tumor were 42 ± 1.7 d, 110 ± 4.7 d and ≥300 d for photons and 44 ± 1.7 d, 80 ± 2.0 d and ≥300 d for ^12^C-ions, respectively. While locally controlled AT1- and HI-tumors regressed completely, tiny nodules remained in case of locally controlled H-tumors. Lack of proliferative activity associated with a fibrotic tissue pattern used as secondary histological endpoint resulted in an increase of TCD_50_ of 10.1 Gy for photons but only 3.2 Gy for ^12^C-ions. The corresponding RBE was 1.80 ± 0.13.

### Structural and functional characterization of the three sublines

**Fig. 2:**
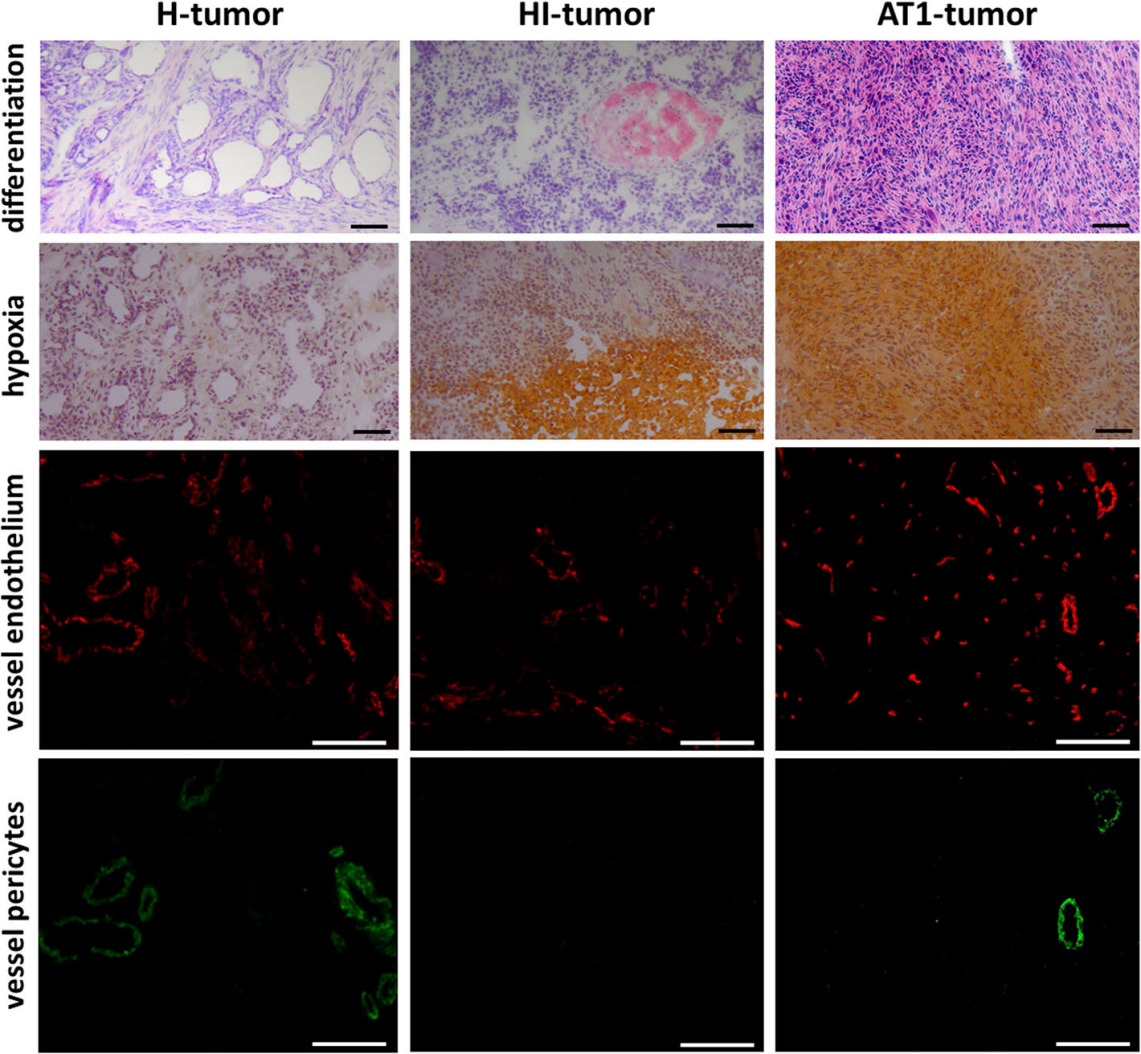
Comparison of histological sections for the H- (left column), HI- (middle column) and AT1-tumor (right column). Structural changes and differentiation level are detected by Hematoxylin / Eosin (H&E) staining (upper row). For visualization of hypoxic areas, pimonidazole was used (brown staining, 2^nd^ row) and cell nuclei were counterstained with Hematoxylin. Vessel endothelium was stained with CD31 antibody in red (3^rd^ row). The maturity of vessels was proven by a pericyte staining using a smooth muscle actin antibody. Magnification: 100× (1^st^ and 2^nd^ row) or 200× (3^rd^ and 4^th^ row). Scale bars are 100 μm

Histological characterization of the three sublines concerning differentiation, hypoxia and vessel density as well as vessel maturity lead to the conclusion that well-differentiated, slow growing H-tumors have more mature vessels with a minor proportion of hypoxia, whereas HI-tumors are characterized by a more diffuse vascular supply and lack of pericytes in most of their vessels. The AT1-tumor vessels consist of mainly tiny capillary structures causing a volume-dependent higher fraction of hypoxia (Fig. 2).

Dynamic [^18^F]FMISO PET of all three untreated tumor sublines showed standardized uptake values (SUV_max_) of 1.33 ± 0.52 in large AT1-tumors and 1.12 ± 0.83 in large HI-tumors. In H-tumors no significant tracer uptake was found (SUV_max_ 0.63 ± 0.16). These characteristics were confirmed by the histological staining with pimonidazole (hypoxic fraction: 62% (AT1), 54% (HI) and 7% (H)). Small tumors did not show a significant uptake at all. The three sublines exhibited differently-shaped time activity curves (TAC). All H-tumors showed a high perfusion-related peak at a few seconds after tracer injection followed by a rapid decrease. HI-tumors reacted much more heterogeneously, with just a small initial peak and a rapid decrease afterwards in most tumors, however, in some HI-tumors a small peak and a positive slope at later time points was found. These two relative similar TAC-shapes could also be detected in the AT1-tumors, however, a third TAC-shape, described by a barely noticeable initial peak and subsequent increasing uptake thereafter was additionally found.

### DNA-index, cell cycle analysis and surface marker expression using flow cytometry

All three tumor sublines gained aneuploidic sub-populations with a high proliferative activity as compared to normal prostate tissue. Ploidy status as well as the number of aneuploidic cells were significantly higher in the AT1-tumors than in the two other tumor lines. In addition, the AT1-tumor always exhibited the largest amount of potential stem cells (i.e. CD24^+^/CD45^−^ cells). Injecting 100 of these potential stem cells subcutaneously into the hind limb of animals resulted in an induction and complete reproduction of tumors with the same histology for the H- and HI-, but not for the AT1-tumor subline. More details on the results of this study can be found in Glowa et al. [15].

### Dose-response studies for HI-tumors with and without clamping

Large HI-tumors were selected for this study because this moderately differentiated tumor exhibited the largest response heterogeneity after photon irradiation. As compared to small tumors, the TCD_50_-values for the large tumors after single dose irradiations were about 30% higher under ambient conditions. For the large tumors, the TCD_50_-value after a single dose of photon irradiation under clamping as compared to ambient conditions was found to be increased by ~15%, while no difference was found for ^12^C-ions and for ^16^O-ions. This, in turn, lead to an increased RBE under hypoxic conditions for both ion types.

### Treatment effects after photon and carbon ion irradiation

**Fig. 3:**
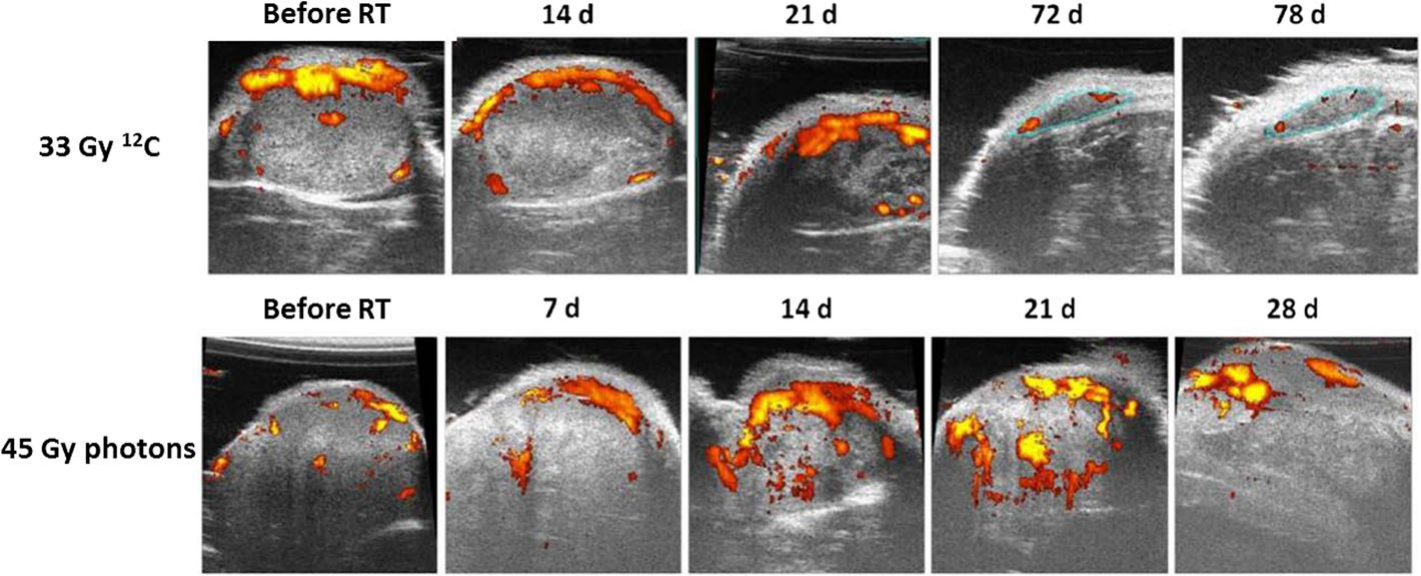
Representative axial images of color-coded power doppler ultrasound overlayed with a B-Mode image showing a middle HI-tumor section before and after selected time points after single doses: yellow and red pixels represent the blood flow. The blue contours after 72 and 78 days in the upper row represent the persisting residual tumor volume of a local controlled HI-tumor. The lower panel describes the time course of a photon treated HI-tumor without local tumor control / with a regrowing tumor showing continuous volume and blood flow increase. After carbon ion irradiation, the tumor volume reduction as well as the decrease in power doppler signal reveals a tremendous decrease in blood flow which is not seen after photon treatment at early time points

Doppler ultrasound imaging in HI-tumors exhibited a decrease in tumor volume as well as in blood flow after single dose treatment with 33 Gy ^12^C-ions and 75 Gy photons, respectively. In these locally controlled tumors, the blood flow decreased to an individual tumor baseline and remained constant thereafter (Fig. 3 upper row). The histologically determined high perfusion early after irradiation was well reflected by the ultrasound measurements. However, tumors treated with a single fractions of subtherapeutic doses (21 Gy ^12^C–ions or 45 Gy photons) showed an increase of blood flow with increasing tumor volume (Fig. 3 lower row). Compared to unirradiated controls, the blood flow decreased after irradiation, but averaged over all animals, there was no significant difference between locally controlled and uncontrolled tumors or between photons and ^12^C-ions in HI-tumors until the onset of clear tumor recurrence.

**Fig. 4:**
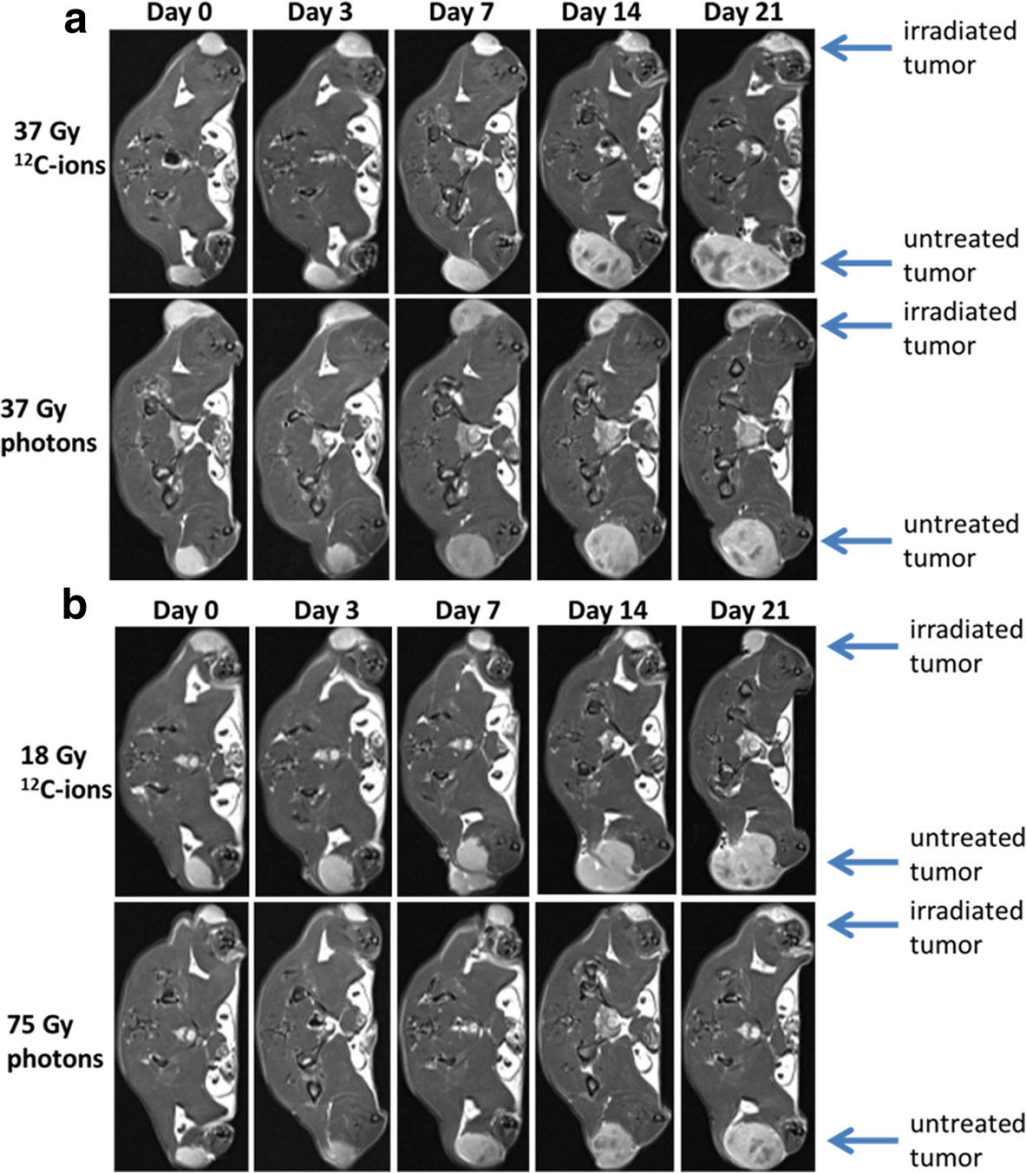
Exemplarily T1-weighted axial MR-images measured 6 min after contrast agent injection. HI-tumors were either treated (upper tumor in each image) or untreated (lower tumors). Tumors were measured before RT (Day 0) and at 4 time points after single doses. Due to the fast growth of untreated tumors, a longer follow-up was not possible. In (**a**) 37 Gy isodoses of photons and ^12^C-ions are compared whereas in (**b**) isoeffective doses with respect to local control at 300 days are shown (18 Gy ^12^C-ions vs. 37 Gy photons and 37 Gy ^12^C-ions vs. 75 Gy photons, respectively). A volume increase was seen only in untreated tumors. The light contrast showed edema, whereas dark volumes are a sign for necrotic areas

HI-tumors were irradiated with single doses using either the same physical doses (37 Gy) or isoeffective doses (18 Gy ^12^C-ions vs. 37 Gy photons and 37 Gy ^12^C-ions vs. 75 Gy photons) of photons and ^12^C-ions, respectively. Necrosis was identified by combining T2-weighted as well as T1-weighted images. After MRI contrast agent injection a more pronounced and faster signal enhancement for both dose levels was seen at 3 and 7 days after irradiation with ^12^C-ions compared to photons (Fig. 4).

**Fig. 5:**
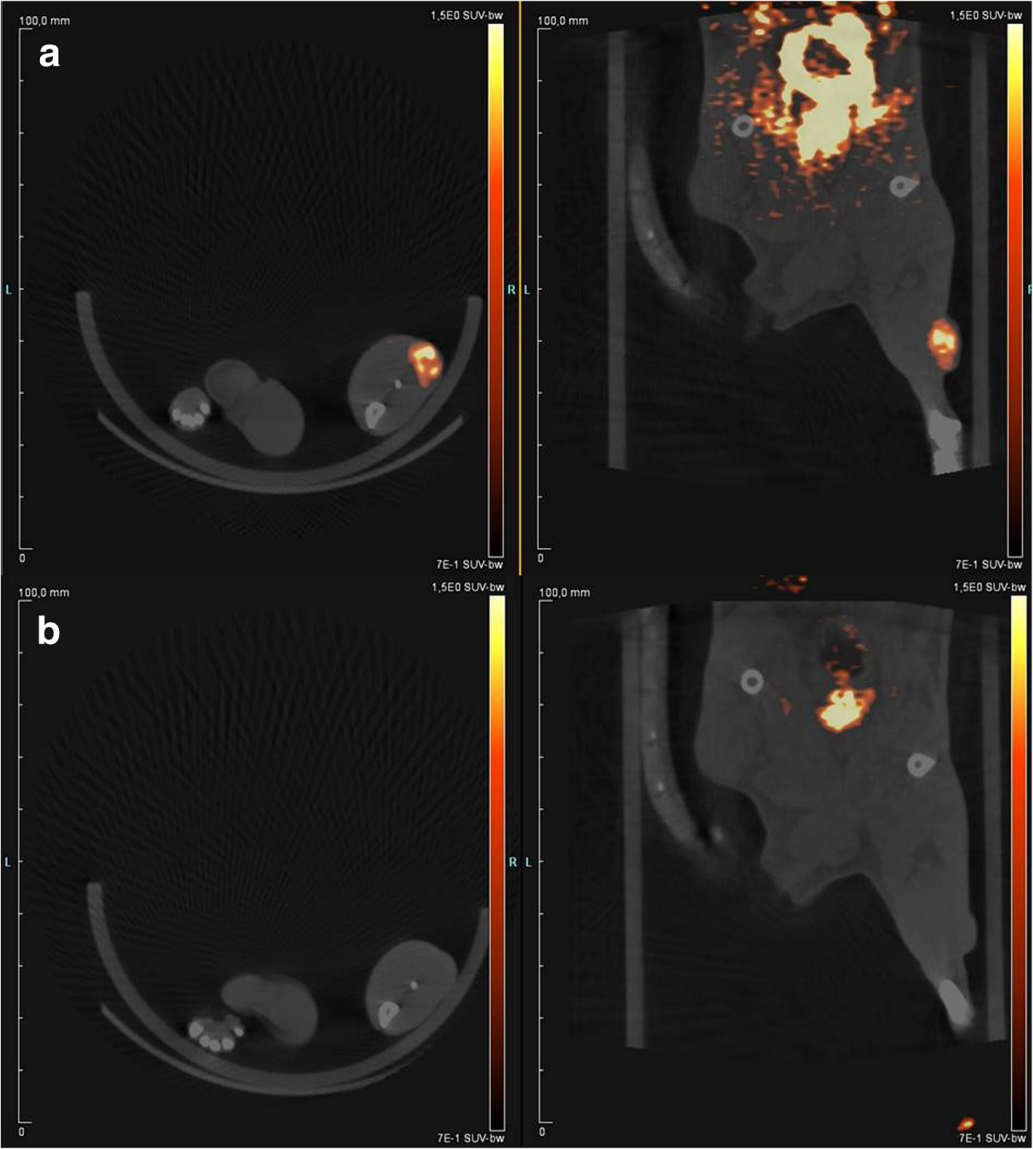
[^18^F]FAZA-PET/CT of a HI-tumor 2 h post tracer injection, before (**a**) and after single fraction of ^12^C-ions (**b**) in axial (left panel) and coronar (right panel) image orientations. The PET tracer is color-coded and overlayed to an aligned CT image. Before RT (**a**), a high tracer uptake was found in the tumor as well as in the bladder due to partly urinary excretion of the tracer. 7 days after RT (**b**), the same tumor showed a tracer uptake in the bladder but not in the tumor

Static [^18^F]FAZA-PET measurements of large HI-tumors before treatment showed an SUV_max_ of up to 1.8 and an increased tumor-to-muscle ratio greater than 3. Independent of the radiation quality, an apparent complete reoxygenation was found with [^18^F]FAZA-PET 7 days after single fraction irradiations (Fig. 5).

Histological analysis revealed a shortened latency time for vessel damage, cell cycle arrest and cell death with a concordant prolonged repair time after carbon ions as compared to photons in all three sublines after single fraction irradiation. In addition, inflammatory activity was markedly increased. Gene expression profiling and molecular analysis confirmed these results. Generally, genes involved in DNA-repair, cell cycle arrest, cell-cell interaction and migration as well as cytokines and caspases were overexpressed. Especially the time-dependent induction of genes was extended after carbon ions.

## Discussion

Preclinical studies in normal tissues are preferentially performed to evaluate potential side effects of carbon ions and to validate RBE-models. In contrast, tumor experiments aim to decipher biological factors, which influence the tumor response differently for photons and ion beams, and to identify, which tumor entities might benefit most likely from high-LET irradiations. In this context, a systematic study was initiated to quantitatively assess the treatment response of three different tumor lines to photons and ^12^C-ions using a local tumor control assay.

In summary, the following clinically relevant results (Fig. 1) were obtained: (i) For photons, a considerably heterogeneous treatment response was found, documented by a broad range of TCD_50_-values for the three tumor-sublines. (ii) For carbon ions, the respective dose-response curves were located much closer to each other. (iii) In addition, the slope of the dose-response curve for each tumor subline was comparable or steeper for ^12^C-ions than for photons, and (iv) the resulting RBE increased with tumor grading (i.e. H vs. HI vs. AT1). This increase of RBE predominantly results from a rise of TCD_50_ with tumor grading in photon treatments while the variation of the treatment response to ^12^C-ions is only small. This supports the conclusion that certain tumor-associated factors might be responsible to render tumors more resistant to photons than to ^12^C-ions. Clearly, these factors are depending on tumor grade. Moreover, also intra-tumoral heterogeneity seems to possess minor impact as documented by the increased slope of the dose-response curve of ^12^C-ions for the very heterogeneous HI-subline as compared to the respective curve for photons. These results allow the conclusion that the response to ^12^C-ions is also less dependent on intra-tumor heterogeneity. Regarding the effectiveness, the highest RBE of ^12^C-ions can be expected for undifferentiated tumors, showing the highest resistance against photon irradiations. A first report on prostate cancer patients in Japan confirmed our results showing very high tumor control rates with reduced toxicity and a comparable 5-year local control rate for carbon ions between low, intermediate and high-risk prostate cancer patients [20].

From a technical point of view, assessment of local control was most difficult in the slow-growing and well differentiated H-tumor because of frequently occurring residual tissue nodules at the end of the follow-up time. This problem was solved by additional histological analysis using lack of proliferative activity within these nodules as secondary endpoint. Interestingly, as the corresponding TCD_50_-values increased for both, photons and ^12^C-ions, there was only a minor difference in RBE and the above conclusion remains unchanged [6].

While this report refers to single dose irradiations only, the identical study was conducted for 2 and 6 fractions, already published for the AT1-tumor [14]. Although still under evaluation for the HI- and the H-tumor, there is a clear trend that fractionation increases the TCD_50_-values in all three tumor cell lines and both irradiation modalities. Again, the shift is larger for photons than for carbon ions, indicating an increasing RBE with decreasing dose per fraction and decreasing differentiation status. The highest RBE for 6 daily fractions (2.67 ± 0.15) was found for the anaplastic AT1-subline [14]. Details on the complete fractionated studies, including the dose dependence of the RBE and the determination of α/β-ratios will be published separately. An interesting side observation of the published study [14] was that in the fast growing AT1-tumor the metastatic rate increased, when the number of fractions raised from 2 to 6. Yet, at least for the given treatment schedules (1, 2 and 6 fractions) the results were not dependent on radiation quality [21].

There is significant evidence in the literature that resistance to photon therapy is associated with both, intrinsic cellular factors conditioned by the evolutionary capacity of cancer phenotypes as well as epigenetic parameters, or the temporal and spatial heterogeneity of the tumor microenvironment caused by structural abnormalities and density of tumor microvessels, dysfunctional blood flow, low pH leading to either chronic or acute hypoxic conditions [22–25].

For further clarification, a detailed structural and functional characterization of all three tumor lines prior to irradiation was undertaken. As highly aneuploidic subpopulations were present in all three tumor lines, the ploidic status was not considered as a relevant tumor-associated intrinsic factor for the differential radiation response [15]. In contrast, differences were detected with respect to putative cancer stem-like cells characterized as CD24^+^/CD45^−^ cells, which were positively tested for the ability to form new tumors in a functional limiting dilution assays (Glowa et al., unpublished data). The fact that stem-cell properties were detected in H- and HI- but not in AT1-tumors needs further analysis which is presently ongoing.

Dramatic differences were found with respect to the structure and quality of the tumor vascularization and in correlation with the tumor microenvironment, inasmuch as a range of differently oxygenated tumors were detected, with the highest hypoxic fraction in the poorly differentiated AT1-tumors and nearly no detectable hypoxia in the well differentiated H-tumors. These results are in line with a previous report on the same tumor model using TOLD-MRI [10]. In addition, the [^18^F]FMISO-TAC-curves in PET were extremely variable between the three tumor sublines indicating also large differences in perfusion [5]. Thus, the investigated tumor-sublines represent a wide range of differently oxygenated tumors allowing dedicated investigation of the role of oxygenation on the radiation response.

To further exploit the role of ^12^C-ions to overcome hypoxia, which is presumably the most important resistance factor in photon therapy, a four-armed dose-response study was performed. Larger moderately differentiated HI-tumors were selected as model tumors because of its proven hypoxia and its extensive heterogeneous treatment response to photons. For larger HI-tumors treated with photons either under ambient or complete hypoxic (clamping) conditions, the detected oxygen enhancement ratio (OER) was clearly below 2, which is in line with previously published in vivo studies [26, 27]. Generally, OERs for single dose irradiations in solid tumors under clamping conditions were found to be lower than in cell culture studies [3, 28], presumably because tumor cells in intact tissues are not only impacted by the intrinsic cellular radioresistance but also by additional factors like cell to cell communication, bystander effects, and the immune response. Moreover, clamping does not only create a transient severe hypoxic state but also reduces the nutrient supply and induces a strong extracellular pressure to the capillaries which might increase secondary tumor cell death and therefore masks the potentially higher OER to some degree. When ^12^C-ions (dose average LET: 65 keV/μm) were applied under identical experimental conditions an up to 15% lower OER was found for larger HI-tumors. The detected decrease of OER for ^12^C-ions is relevant and if confirmed in patients would increase effectiveness dramatically. With this respect, the only available study, which compares the impact of tumor oxygenation for ^12^C-ions and photons in patients, is inconclusive [29].

A detailed comparison with the previous dose-response experiments for the small tumors, however, turned out to be difficult as the TCD_50_-values after photon and ^12^C-ion irradiations under non-clamping conditions were found to be substantially higher as compared to the previously investigated small tumors. This suggests that larger tumors are not only associated with an increased number of tumor cells but also that volume-dependent alterations of the tumor micromilieu might play a role. To investigate this hypothesis in more detail, the clamping experiments are currently repeated for the small tumors within a new project and a comparison of the response of small and large tumors will be published separately when the results are available.

Radioresistance of tumors due to hypoxia is clinically of highest relevance as oxic tumors have a much higher disease-free survival than hypoxic tumors, as has been shown in head and neck cancer patients [30]. Therefore, the observed reduction of the OER for ^12^C-ions is a very important finding for the treatment of hypoxic tumors. In a first patient cohort treated with carbon ions, Japanese colleagues verified a smaller OER of ^12^C-ions in uterine cancer [29] and our findings in the experimental prostate carcinomas confirm this, however, further analyses are necessary. Although the obtained promising OERs for ^12^C-ions might in principal be used to overcome radioresistance evoked by severe hypoxia, the dependence of OER on LET is still an open question. In vitro the OER for high LET irradiations decreases with increasing LET and is expected to be negligible at LETs higher than 200 keV/μm [31]. Our preliminary results based on dose-response studies with oxygen ions (dose average LET: 101 keV/μm) using the same tumor model also indicates a small OER near to 1.

Finally, structural and functional assessments were performed in HI-tumors to decipher some of the biological mechanisms, responsible for the differential effects of ^12^C-ions and photons. In-house synthetized [^18^F]FAZA in combination with static PET measurements in a dedicated small animal scanner (Inveon Micro-PET/SPECT/CT (Siemens Medical Solutions, Knoxville, USA)) has been established in larger HI-tumors. A significant tracer-uptake prior to treatment followed by a clear reduction 1 week after irradiation was found for photons as well as for ^12^C-ions in a first pilot study. The hypoxia imaging tracer [^18^F]FAZA is a well-established alternative to the first generation tracer [^18^F]FMISO and is known to correlate well with both, autoradiography and the hypoxia marker pimonidazole [32]. Yet, results so far are reported to be ambivalent. No significant general reoxygenation was seen for SiHa cervix tumors in mice after fractionated irradiations with 10 or 25 Gy photons [32], while reoxygenation has been shown after 2 weeks of fractionated radiotherapy for patients with head and neck cancer in some but not in all cases [33]. Radiation-induced reoxygenation seems to depend on tumor type and on the intrinsic characteristics of the individual tumor. Tumor cell inactivation, tissue shrinkage, vessel damage and altered perfusion are biological factors associated with oxygenation changes after irradiation. A very striking observation was the extremely fast emerging of vascular disruptions already 18 h after single doses of carbon ions, while similar changes were delayed after photon irradiations. Nevertheless, a clear correlation between vessel integrity and hypoxia or reoxygenation is currently missing.

In spite of existing vascular structures, oxygen delivery to neoplastic and stromal cells is frequently reduced or even abolished by increased vessel distances, severe structural abnormalities of tumor microvessels, disturbed microcirculation and increased interstitial pressure. This can lead to false negative results in PET measurements as the tracer may not reach the hypoxic regions. To independently assess functional microenvironmental disturbations, measurements with doppler ultrasound as well as DCE-MRI were performed. Ultrasound measurements revealed a high blood flow followed by a rapid and dramatic decrease after carbon ions and later a steady state in the first 4 weeks after irradiation. Besides, this initial effect no other significant variation was detected between the two radiation modalities as well as for different dose levels. A further refinement of these results is expected from photoacoustic imaging [34], which allows assessing the oxygen saturation of tumor vessels based on optical detection of oxy- and deoxyhemoglobin. Similar to the ultrasound measurements, preliminary results of DCE-MRI revealed a faster and higher contrast enhancement after single doses of ^12^C-ions as compared to photons, which is in line with the more prominent vessel damage observed on the histological level. This first assessment is based on a qualitative rather than quantitative data analysis and a more detailed investigation using pharmacokinetic modeling to extract perfusion-related tissue parameters is ongoing.

## Conclusions

Although the picture of the differential response to photon and ^12^C-ion irradiation is not yet fully completed, the described radiobiological research program has systematically documented the higher efficacy of ^12^C-ion therapy in a syngeneic experimental tumor model. It has been shown that tumor-associated resistance factors can be partly overridden by high-LET ion beam therapy. Moreover, the results provide evidence that the increased effectiveness of high-LET radiation is related to tumor-associated factors, preferentially those which are responsible to render tumors more resistant to conventional photon treatments. Dose-response studies revealed that hypoxia is one of the dominant radioresistance factors and that its impact can be reduced by high-LET ion beams on a clinically relevant level. This could potentially improve the probability of local tumor control relative to conventional photon treatments. Pronounced vascular disruptions associated with enhanced cell inactivation together with a rapid disturbance of the tumor microenvironment were identified as the most striking mechanistic effects of carbon ions and these results served as starting point for various ongoing research projects.

## Abbreviations

[^18^F]FAZA: [^18^F]fluoroazomycin arabinoside
[^18^F]FMISO: [^18^F]fluoromisonidazole
^12^C-ions: Carbon ions
^16^O-ions: Oxygen ions
BrdU: Bromodesoxyuridine
DCE-MRI: Dynamic contrast enhanced magnetic resonance imaging
DFG: German Research Foundation
FFPE: Formalin-fixed paraffin-embedded
H&E: Hematoxylin/Eosin staining
LET: Linear energy transfer
OER: Oxygen enhancement ratio
PET: Positron-Emission-Tomography
PMMA: Polymethyl methacrylate
RBE: Relative biological effectiveness
SOBP: Spread-out Bragg-peak
SUV: Standard uptake value
TAC: Time activity curve
TCD_50_-value: Irradiation dose with 50% tumor control probability
